# Mice with a Brd4 Mutation Represent a New Model of Nephrocalcinosis

**DOI:** 10.1002/jbmr.3695

**Published:** 2019-03-04

**Authors:** Caroline M Gorvin, Nellie Y Loh, Michael J Stechman, Sara Falcone, Fadil M Hannan, Bushra N Ahmad, Sian E Piret, Anita AC Reed, Jeshmi Jeyabalan, Paul Leo, Mhairi Marshall, Siddharth Sethi, Paul Bass, Ian Roberts, Jeremy Sanderson, Sara Wells, Tertius A Hough, Liz Bentley, Paul T Christie, Michelle M Simon, Ann‐Marie Mallon, Herbert Schulz, Roger D Cox, Matthew A Brown, Norbert Huebner, Steve D Brown, Rajesh V Thakker

**Affiliations:** ^1^ Academic Endocrine Unit Oxford Centre for Diabetes, Endocrinology, and Metabolism Radcliffe Department of Medicine University of Oxford Oxford UK; ^2^ Institute of Ageing and Chronic Disease University of Liverpool Liverpool UK; ^3^ Translational Genomics Group Institute of Health and Biomedical Innovation School of Biomedical Sciences Queensland University of Technology (QUT) at Translational Research Institute Brisbane Australia; ^4^ Mary Lyon Centre and Mammalian Genetics Unit Medical Research Council Harwell Institute Harwell UK; ^5^ Department of Cellular Pathology Royal Free Hospital London UK; ^6^ Department of Cellular Pathology John Radcliffe Hospital Oxford UK; ^7^ Max‐Delbrück‐Center for Molecular Medicine Berlin Germany

**Keywords:** NEPHROLITHIASIS, NEPHROCALCINOSIS, MOUSE MODEL, BRD4 MUTATION

## Abstract

Nephrolithiasis (NL) and nephrocalcinosis (NC), which comprise renal calcification of the collecting system and parenchyma, respectively, have a multifactorial etiology with environmental and genetic determinants and affect ∼10% of adults by age 70 years. Studies of families with hereditary NL and NC have identified >30 causative genes that have increased our understanding of extracellular calcium homeostasis and renal tubular transport of calcium. However, these account for <20% of the likely genes that are involved, and to identify novel genes for renal calcification disorders, we investigated 1745 12‐month‐old progeny from a male mouse that had been treated with the chemical mutagen *N*‐ethyl‐*N*‐nitrosourea (ENU) for radiological renal opacities. This identified a male mouse with renal calcification that was inherited as an autosomal dominant trait with >80% penetrance in 152 progeny. The calcification consisted of calcium phosphate deposits in the renal papillae and was associated with the presence of the urinary macromolecules osteopontin and Tamm‐Horsfall protein, which are features found in Randall's plaques of patients with NC. Genome‐wide mapping located the disease locus to a ∼30 Mbp region on chromosome 17A3.3‐B3 and whole‐exome sequence analysis identified a heterozygous mutation, resulting in a missense substitution (Met149Thr, M149T), in the bromodomain‐containing protein 4 (*BRD4*). The mutant heterozygous (*Brd4^+/M149T^*) mice, when compared with wild‐type (*Brd4^+/+^*) mice, were normocalcemic and normophosphatemic, with normal urinary excretions of calcium and phosphate, and had normal bone turnover markers. *BRD4* plays a critical role in histone modification and gene transcription, and cDNA expression profiling, using kidneys from *Brd4^+/M149T^* and *Brd4^+/+^* mice, revealed differential expression of genes involved in vitamin D metabolism, cell differentiation, and apoptosis. Kidneys from *Brd4^+/M149T^* mice also had increased apoptosis at sites of calcification within the renal papillae. Thus, our studies have established a mouse model, due to a *Brd4* Met149Thr mutation, for inherited NC. © 2019 American Society for Bone and Mineral Research.

## Introduction

Renal calcification may occur in the kidney parenchyma (nephrocalcinosis [NC]) or the collecting system (nephrolithiasis [NL], ie, kidney stones).^1^, ^2^, ^3^, ^4^ NL and NC affect ∼10% of the adult Western population by the age of 70 years,^1^, ^2^, ^3^ although such distinctions may be difficult in patients who may develop both NL and NC, and it has been reported that >50% to 70% of patients with kidney stones will have NC.^5^ The renal calcification in NL and NC results from deposition of calcium oxalate and calcium phosphate,^4^ which has a multifactorial etiology that involves environmental and genetic causes and may also be associated with several endocrine and metabolic disorders, including primary hyperparathyroidism, hypercalciuria, renal tubular acidosis, cystinuria, low urinary volume, and hyperoxaluria.^1^, ^6^ Hypercalciuria is the commonest metabolic abnormality associated with NL and NC; however, around 30% of individuals with kidney stones have been reported to have no obvious underlying metabolic defect (idiopathic NL).^1^, ^6^, ^7^, ^8^ NL, NC, and hypercalciuria are likely to have a genetic basis as up to 65% of kidney stone patients have been reported to have an affected family member^9^, ^10^ and twin studies have estimated that the heritability of hypercalciuria^11^ and kidney stones^12^ is >50% and >55%, respectively. Moreover, studies of families with rare monogenic disorders associated with hypercalciuric NL and NC, such as Bartter's syndrome, Dent's disease, autosomal dominant hypocalcaemia, and distal renal tubular acidosis, have identified mutations in >30 genes involved in the regulation of calcium transport (Supplemental Table S1).^3^, ^13^, ^14^ Furthermore, genome‐wide association studies and targeted sequencing of genes with known roles in calcium and vitamin D metabolism have reported associations for NL and NC with common sequence variants in >10 additional genes (Supplemental Table S1).^2^, ^15^, ^16^, ^17^ However, these account for only ∼15% to 20% of cases,^3^, ^13^ and the identification of further monogenic causes of NL and NC are limited by the availability of large families. To overcome this and to facilitate further positional cloning studies to discover other genes regulating renal calcification, we undertook an X‐ray screen of first‐generation (G1) offspring of mice treated with the chemical mutagen *N*‐ethyl‐*N*‐nitrosourea (ENU),^18^ for renal opacities, with the aim of identifying mice with renal calcium deposits, as calcium‐containing stones are the most common type of kidney stones. ENU is an alkylating agent that causes germline point mutations with a frequency as high as 0.015 mutations/locus.^19^ Such mutations may result in loss‐of‐function and hypomorphic, hypermorphic, and dominant‐negative changes in protein function, thus making the ENU mouse mutagenesis resource a useful tool for generating novel genetic models of human disease.^18^, ^19^ Here, we describe a mouse model for autosomal dominant renal calcification, designated RCALC type 1 (RCALC1), identified from this ENU screen, which is associated with a Met149Thr missense mutation in the gene encoding bromodomain containing protein 4 (*Brd4*).

## Materials and Methods

### Experimental animals

**Figure 1:**
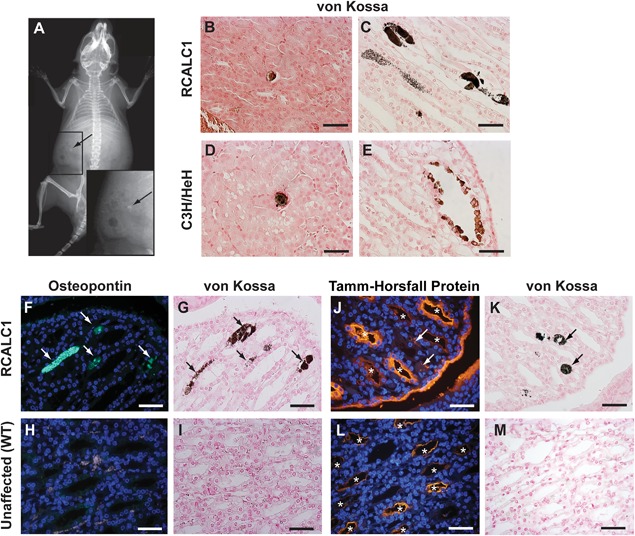
Identification and characterization of RCALC1 mice. (*A*) Radiograph of the 12‐month‐old G1 founder male mouse showing renal opacity (arrow). Inset: an enlarged image of the renal opacity. (*B*, *C*) Presence of renal calcification (brown) in (*B*) the cortex and (*C*) the interstitial regions of the renal papilla in kidney sections of G2 offspring of the G1 founder male stained by von Kossa. (*D*, *E*) Renal calcification (brown) in (*D*) the cortex and (*E*) the collecting duct cells in kidney sections of wild‐type (WT) C3H/HeH inbred mice, stained by von Kossa. (*F–M*) Immunofluorescence and von Kossa staining of serial sections of kidneys from an RCALC1 mouse and an unaffected WT littermate. (*F–I*) Osteopontin (OPN) and von Kossa staining, showing co‐localization of OPN staining (green, white arrows) with areas of interstitial calcification in RCALC1 mice (black arrows), that was not observed to occur in WT mice. (*J–M*) Tamm‐Horsfall protein (THP) and von Kossa staining, showing the presence of THP (orange, asterisks) in renal thick ascending limbs of RCALC1 and WT mice. THP immunostaining and von Kossa staining, corresponding to intersititial calcification, co‐localize (arrow) in RCALC1 mice. Scale bars = 50 μm.

ENU‐treated G0 BALB/cAnNCrl male mice were mated to C3H/HeH (C3H) females to produce G1 progeny. Male G1 offspring were X‐rayed for renal opacities and sperm archived, as previously described.^20^, ^21^ Archived sperm from the founder male with renal calcification (Fig. 1 *A*) was used for in vitro fertilization (IVF) of C3H oocytes to derive G2 animals for inheritance testing and genetic mapping studies.^20^ Mice were fed on a standard diet (Rat and Mouse number 3, Special Diet Services, Essex, UK) that contained 1.15% calcium, 0.58% phosphate, and 4089 IU/kg of vitamin D, and provided with water *ad libitum*.^22^ All animal studies were carried out using guidelines issued by the UK Medical Research Council in Responsibility in Use of Animals for Medical Research (July 1993) and UK Home Office project licence numbers (30/2250 and 30/2752).

### Metabolic cage studies, plasma, and urine biochemistry

Sixteen‐week‐old G2 mice were individually housed in metabolic cages (Techniplast, Louviers, France) for 5 days with free access to food and water.^23^ Mice were weighed before and after the study, and food and water intake were monitored. Twenty‐four‐hour urine samples were collected in the presence of sodium azide, and blood samples were collected from lateral tail vein or the internal jugular vein in lithium heparin Microvette tubes (Sarstedt, Leicester, UK) after terminal anesthesia, as previously described.^23^ Plasma and/or urine were appropriately analyzed for sodium, potassium, chloride, total calcium, phosphate, urea, glucose, creatinine, magnesium, urate, citrate, total protein, albumin, and alkaline phosphatase activity on a Beckman Coulter AU680 analyzer, as previously reported.^21^, ^22^ Plasma calcium was adjusted for variations in albumin concentrations using the formula: (plasma calcium (mmol/L) − [(plasma albumin (g/L) − 30) × 0.02]), as reported.^22^ Plasma parathyroid hormone (PTH) was measured using a two‐site ELISA specific for mouse intact PTH (Immutopics, San Clemente, CA, USA).^22^ Plasma concentrations of 25‐hydroxyvitamin D (25OHD) and procollagen type 1 N‐terminal propeptide (P1NP) and also urinary concentrations of serum C‐telopeptide (CTx) were measured by enzyme immunoassay (EIA) (Immunodiagnostic Systems, Bolden, UK).^24^, ^25^ Urine pH was measured using a pH 211 meter (Hanna Instruments, Smithfield, RI, USA).^26^ A Kruskal‐Wallis test was undertaken for multiple comparisons, and any significant differences identified were further assessed using the Dunn's test for nonparametric pairwise multiple comparisons. All analyses were undertaken using GraphPad Prism software (GraphPad, La Jolla, CA, USA), and a value of *p* < 0.05 was considered significant.

### Kidney histology and immunofluorescence

Kidneys were dissected from 16‐ to 18‐week‐old mice, halved, fixed in 10% neutral‐buffered formalin overnight, and embedded in paraffin wax. Then 4‐μm serial sections were prepared and stained with hematoxylin and eosin (H&E), and von Kossa, which reacts with the phosphate moiety of calcium phosphate,^27^ as described previously.^20^ Images were collected on a Nikon Eclipse E400 microscope (Kingston‐upon‐Thames, UK), equipped with a Nikon DXM1200C digital camera. Slides were examined by two independent observers, by light microscopy using 10× and 20× objective lenses to confirm the presence of renal calcification. Each observer was blinded to the experimental group, and in cases in which there was disagreement, a third investigator reviewed the slide and made the final decision. Coincident calcification on at least two serial sections was accepted as positive, and the H&E‐stained sections were then examined under polarized light for possible birefringence, which would indicate the presence of calcium oxalate crystals in the depositions,^27^ using a Zeiss Axiocam MRc (Cambridge, UK) camera, with a pixel size of 6.7 µm and a 1300 × 1030 pixel field of view equivalent to an area of 8.7 mm × 6.9 mm, that was adapted for polarized light microscopy with plan semi‐apochromat 10×/NA 0.3 and 20×/NA 0.35 LWD objectives.

Pizzolato staining to detect calcium oxalate was performed as described.^28^ Briefly, 4‐μm kidney serial sections were deparaffinized, rehydrated, then incubated in a solution of equal quantities of 5% silver nitrate and 30% hydrogen peroxidase under a 60‐watt light bulb. Sections were then counterstained with nuclear fast red. Osteopontin immunohistochemistry was performed on deparaffinized, rehydrated 4‐μm kidney sections after antigen retrieval using an anti‐osteopontin rabbit polyclonal antibody (L‐175, kind gift from LW Fisher, NIH, Bethesda, MD, USA),^29^ followed by an Alexa Fluor 488‐conjugated donkey anti‐rabbit antibody (Molecular Probes, Hilden, Germany). Tamm‐Horsfall protein (THP) staining was performed using sheep anti‐THP (Biodesign International, Saco, ME, USA), followed by a secondary detection using Cy3‐conjugated donkey anti‐sheep antibody (Jackson ImmunoResearch, Newmarket, UK). Controls were performed without a primary antibody and showed no staining.

Terminal deoxynucleotidyl transferase dUTP nick end‐labeling (TUNEL) to detect apoptotic cells was performed using the ApopTag Fluorescein In Situ Apoptosis Detection Kit (Millipore, Watford, UK) according to the manufacturer's instructions. Stained sections were mounted in Vectashield containing DAPI (Vector Labs, Peterborough, UK) visualized by epifluorescence microscopy and a Nikon DXM1200C digital camera. The NIS‐Elements BR 2.30 software was used to estimate the number of TUNEL‐positive cells per 1000 DAPI‐positive cells. Statistical comparisons for TUNEL staining were performed using the Fisher's exact test and chi‐square test with Yate's correction.

### Genetic mapping

Genomic DNA was isolated from tail biopsies using the Gentra PureGene kit (Qiagen, Manchester, UK) and genome‐wide scans performed by pyrosequencing^30^ using a panel of 59 informative single‐nucleotide polymorphisms (SNPs) distributed across 19 autosomes, at 20‐ to 30‐cM intervals. Polymorphic positions were analyzed using a PSQ‐96 system (Qiagen).^20^ Chromosomal linkage was verified by noninheritance of BALB/c alleles of SNPs of interest in 13 unaffected littermates. G2 and G3 mice were genotyped for additional markers within the critical region.

### Exome sequence analysis

Sequencing libraries were constructed using the NimbleGen Kit (Roche, West Sussex, UK) and libraries combined in pools of six for targeted capture, using the SeqCap EZ Mouse Exome SR v2.2 (target regions available from ftp://ftp.jax.org/Genome_Biology_mouse_exomes). Libraries were assessed pre‐ and post‐capture for quality and yield, using a High Sensitivity DNA assay (Agilent, Santa Clara, CA, USA) and Library Quantification Kit (Kapa Biosystems, Gillingham, UK). Massive parallel sequencing was performed with six samples per flow cell lane, using the HiSeq2000 platform and SBS reagents (Illumina, San Diego, CA, USA) to generate 100‐bp paired‐end reads. Illumina Data Analysis Pipeline software (CASAVA 1.8.1) was used for initial base calling and data multiplexing. Illumina reads were mapped to the mouse genome (mm9) using the Burrows‐Wheeler Aligner (BWA)_v2^31^ with the default parameters. Single‐nucleotide variant (SNV) cells were made using a customized version of The Genome Analysis Toolkit (GATK)^32^ with default parameters. Several triaging steps were made to reduce false positives.^33^ The 17 Mouse Genome data set^34^ was used to filter inbred SNP sites from the RCALC1 SNV data set, and common sites were removed from further investigation. The remaining SNVs were further filtered by removing sites with an allele frequency <35% and >80%, a read depth <3, and a quality score <200. The final RCALC1 SNV data set was annotated with NGS‐SNP.^35^

### DNA sequence analysis, protein sequence alignment, and protein prediction

Variants were validated in G2 mice by Sanger DNA sequencing, using appropriate gene‐specific primers (Sigma, Gillingham, UK), followed by dideoxynucleotide sequencing using the BigDye Terminator v3.1 Cycle Sequencing Kit (Life Technologies, Carlsbad, CA, USA) and an automated detection system (ABI3730 capillary sequencer; Applied Biosystems, Carlsbad, CA, USA).^36^ Protein sequences were aligned using ClustalW.^20^ MutationTaster (http://www.mutationtaster.org/) was used to predict the effects of mutations.^37^ BRD4 modeling was undertaken using the BRD4 structure in complex with an extracellular signal‐regulated kinase 5 inhibitor (PDB: 5LRQ), and figures prepared using the PyMOL Molecular Graphics System, Schrodinger (https://pymol.org/).

### RNA extraction, cDNA expression profiling, and quantitative RT‐PCR (qRT‐PCR)

Total RNA was extracted from whole kidney samples taken from >16‐week‐old RCALC1, or parental wild‐type (WT) BALB/c and C3H mice (*n* = 4 mice per group) using Trizol reagent (Invitrogen, Carlsbad, CA, USA). After DNaseI‐treatment, RNA was purified using an RNeasy Mini Kit (Qiagen). Nine micrograms of total RNA was used for first‐ and second‐strand cDNA synthesis using the One‐Cycle cDNA Synthesis Kit (Affymetrix, High Wycombe, UK) according to the manufacturer's instructions. Biotinylated cRNA was synthesized using the Genechip IVT Labelling Kit (Affymetrix). Fifteen micrograms of fragmented cRNA was hybridized for 16 hours at 45°C to Mouse Genome 430 2.0 arrays (Affymetrix). After hybridization, arrays were washed and stained with streptavidin‐phycoerythrin in the Affymetrix Fluidics Station 450 and scanned using the GeneChip Scanner 3000 7G. The image data were analyzed with GCOS 1.4 using Affymetrix default analysis settings and global scaling as a normalization method. The data set of the arrays were normalized using Robust Multi‐chip Average algorithm in respect to the sequence‐specific probe affinities.^38^ Probe sets with marginal expression (maximum native signal <50) were removed and the remaining 17,811 probe sets were analyzed using the F‐test. After ANOVA false discovery rate (FDR) multiple testing corrections,^39^ individual differences between the data sets were investigated using the least significant difference (LSD) post hoc test. Genes with >1.5‐fold difference in expression (in the same direction) versus both parental strains were selected for further investigation. qRT‐PCR reactions were performed in kidney samples from 4 to 5 mice using the QuantiTect SYBR Green Kit (Qiagen) and a Rotorgene 5 (Qiagen), as previously described.^40^ All qRT‐PCR test samples were normalized to the geometric mean of three housekeeper genes (cyclin D1 [*Ccnd1*], cyclin D2 [*Ccnd2*], and glyceraldehyde 3‐phosphate dehydrogenase [*Gapdh*]), as described previously).^40^ All primers were obtained from Quantitect (Qiagen). Threshold cycle (C_T_) values were obtained from the start of the log phase on Rotorgene Q Series Software and C_T_ values analyzed in Microsoft Excel 97–2010 using the Pfaffl method.^40^, ^41^ Data for RCALC1 mice were expressed relative to wild‐type littermates, which were set at 1. Statistical analyses were performed using the Student's *t* test.

### Statistical analysis

Statistical analyses were performed using the Kruskal‐Wallis test, Dunn's test for nonparametric pairwise multiple comparisons, Fisher's exact test, chi‐square test with Yate's correction, Student's *t* test, the F‐test, and LSD post hoc tests.

## Results

### Identification and characterization of mice with autosomal dominant renal calcification (RCALC1)

A radiological screen of 1745 12‐month‐old G1 male offspring of ENU‐treated BALB/c males and wild‐type (WT) C3H/HeH (C3H) females was performed to detect renal opacities. This identified a male with a single opacity in the region of the left kidney (Fig. 1 *A*). To confirm genetic transmission of the phenotype of renal calcification, 152 second‐generation (G2) backcross mice (69 males, 83 females) were derived by in vitro fertilization (IVF) of WT C3H oocytes using sperm archived from this founder G1 male with renal calcification. These G2 mice were assessed at 16 to 18 weeks of age by renal histology, as renal opacities could not be detected by X‐ray in mice of this age range. Renal histology was performed using sections stained with H&E (Supplemental Fig. S1) and von Kossa stain (Fig. 1 *B–E*), which specifically reacts with the phosphate moiety of calcium phosphate deposits,^27^ and polarized light microscopy to assess for birefringence due to calcium oxalate crystals (Supplemental Fig. S1). This approach was used because calcium phosphate and calcium oxalate crystals can be distinguished by their tinctorial properties.^27^ Thus, on H&E‐stained sections, calcium phosphate deposits may appear blue or purple (Supplemental Fig. S1), whereas calcium oxalate deposits are translucent; sections treated with von Kossa stains reveal calcium phosphate but not calcium oxalate crystals as dark brown or black deposits (Fig. 1 *B–E*, Fig. S1); and with only calcium oxalate but not calcium phosphate, crystals are birefringent under polarized light.^27^ This analysis showed that the renal calcification in the G2 mice consisted of calcium phosphate, as the deposits were blue‐purple on H&E sections (Supplemental Fig. S1) and deep brown‐black on von Kossa‐stained sections (Fig. 1 *B*, *C* and Supplemental Fig. S1). Moreover, the renal calcification was not birefringent under polarized light (Fig. S1), thereby indicating that it did not contain calcium oxalate crystals, and this was confirmed by use of Pizzolato staining and observation of a lack of brown‐black deposits^28^ (Supplemental Fig. S1).

The calcification, due to calcium phosphate deposits, was observed to occur in the renal cortex and papilla of the G2 mice (Fig. 1 *B*, *C*). However, spontaneous renal calcification has been reported to occur in WT C3H mice,^42^, ^43^ and to minimize confounding influences on the phenotype of renal calcification from the WT C3H strain, we further characterized the locations of the renal calcification by von Kossa staining using kidney sections from WT C3H mice at 16 weeks of age (10 males, 10 females). In the WT C3H mice, the renal calcification was found to be present within the cortex of all 20 pairs of kidneys, but calcification within the renal papilla was found to be present in only one mouse (ie, 5% of mice) (Fig. 1 *D*, *E*). This calcification in the renal papilla of the WT C3H mouse was identified within the intracellular compartment of collecting duct cells (Fig. 1 *E*). Therefore, G2 mice with calcification of the renal cortex or intracellular collecting duct cells were classified as being unaffected, as the calcification was not different from that observed in the WT C3H mice, and G2 mice having renal calcification specifically within the interstitial regions of the renal papilla (Fig. 1 *C*) were classified as having the RCALC1 phenotype. Using these criteria, 70 (26 males, 44 females) of the 152 G2 mice (46%) were found to have calcification within the interstitial regions of the renal papilla, consistent with an autosomal dominant disorder, in which ∼50% of progeny would be affected. In addition, the ratio of affected males to affected females was not significantly different to the expected 1:1 ratio of affected males (M) and females (F) (26 M:44 F versus 35 M:35 F, chi‐square test, *p* = 0.125) for an autosomal dominant disorder. The ratio of WT (ie, unaffected) males/females (39 M to 43 F) was also not significantly different from the expected 1:1 ratio (41 M:41 F, chi‐square test, *p* = 0.871), for an autosomal dominant trait.

The RCALC1 phenotype has similarities to human NC, which includes the interstitial deposition of calcium phosphate in the renal papillae.^4^, ^44^, ^45^ For example, high‐resolution Fourier transform infrared microspectroscopy and electron diffraction studies have revealed that calcium phosphate crystals are the major component of Randall's plaque, which are the areas of the renal papillae that contain interstitial calcium phosphate deposits that provide a nidus of urothelial surface for calcium oxalate deposition.^4^, ^6^, ^16^, ^46^, ^47^ Moreover, immunohistological examinations of renal biopsies from patients with kidney stones have shown the presence of the urinary macromolecules osteopontin and THP in association with Randall's plaques,^44^, ^45^, ^48^ and to explore the similarities between the RCALC1 and human NC histological phenotypes, we therefore investigated serial sections of kidneys from the RCALC1 mice and WT littermates for such abnormalities using immunofluorescence to detect osteopontin and THP and von Kossa staining to detect calcification. This revealed the presence of osteopontin in the areas of interstitial calcification within the renal papillae of RCALC1 mice (Fig. 1 *F*, *G*), and this was absent in the renal papillae of WT littermates (Fig. 1 *H*, *I*) (*n* = 4; 2‐tailed Fisher's exact test, *p* < 0.01). Moreover, THP, which was found to be present in the thick ascending limb of Henle in RCALC1 and WT kidneys (Fig. 1 *J*, *L*) was also localized to the interstitial space of the renal papilla in RCALC1 mice (Fig. 1 *J*), where it co‐localized with calcified areas that were revealed by von Kossa staining (Fig. 1 *J*, *K*). Interstitial THP immunostaining was not detected in the renal papilla of WT mice (Fig. 1 *L*, *M*). Therefore, RCALC1 mice have calcification due to calcium phosphate deposition of the renal papilla, which contains osteopontin and THP, and these findings are similar to those observed in patients with NC.^4^, ^46^

### Mapping of the *RCALC1* locus to chromosome 17A3.3‐B3 and identification of the *Brd4* missense mutation by whole‐exome sequence analysis

**Figure 2:**
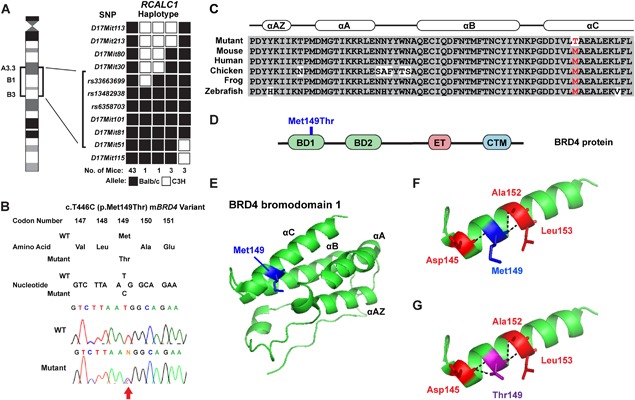
Identification of the *Brd4*‐Met149Thr mutation in RCALC1 mice. (*A*) Haplotype analysis of 51 G2 mice. Each box represents the genotype for the polymorphic locus. Filled box = BALB/c allele; open box = C3H allele. The number of mice that had each haplotype is indicated below each column. Analysis localized the *RCALC1* locus to a ∼15 Mbp region on mouse chromosome 17, flanked by *rs33663699* and *D17Mit51*. (*B*) DNA sequence analysis of *Brd4* in DNA extracted from an unaffected (wild type [WT]) and an RCALC1 mouse (mutant) confirmed the whole‐exome analysis result of a heterozygous T‐to‐C transition in codon 149 in RCALC1 mice that was predicted to result in a missense change of a WT methionine (Met [M]) to a mutant threonine (Thr [T]). (*C*) Multiple protein sequence alignment of residues comprising the αAZ, αA, αB, and αC helices that form bromodomain 1 (BD1) of BRD4 in five species. Conserved residues are shown in gray, and WT (M) and mutant (T) residues in red. (*D*) Domain structure of human BRD4 protein, which comprises two bromodomains (BD1 and BD2), an extraterminal (ET) domain, and a carboxy‐terminal motif (CTM). (*E*) Structure of the human BRD4 bromodomain 1 (PDB:5LRQ) with Met149 indicated in blue. (*F*) Close‐up view of the WT Met149 residue showing backbone polar contacts with three residues, Asp145, Ala152, and Leu153. (*G*) The mutant Thr149 (purple) retains these contacts but forms an additional contact with residue Asp145, which may reduce flexibility of the BRD4 protein and thereby affect its function.

Genome‐wide analysis using a panel of 59 informative SNPs and DNA from 13 G2 mice with renal papillary calcification (the RCALC1 phenotype) mapped the *RCALC1* locus to a ∼30 Mbp region on chromosome 17 A3.3‐B3 between *rs33663699* and *D17Mit115* (LOD = 3.91, 0% recombination) (Fig. 2 *A*). Further mapping studies using 11 additional genetic markers (8 microsatellite markers and 3 SNPs) within this candidate interval in an additional 38 G2 RCALC1 mice (total number of 51 mice studied) revealed the renal calcification phenotype co‐segregated with BALB/c alleles between *rs33663699* and *D17Mit51* (Fig. 2 *A*). Co‐segregation of the RCALC1 phenotype with markers in this chromosome 17A3.3‐B3 interval was confirmed in up to four generations. Finally, 26 of the 152 G2 mice (17%) with the BALB/c haplotypes for the chromosome 17 markers were phenotypically normal (ie, did not have renal papillary calcification), thereby indicating an 83% penetrance for this phenotype in >16‐week‐old G2 mice.

The interval between *rs33663699* and *D17Mit115* that encompasses the *RCALC1* locus contains more than 500 genes, and to identify the RCALC1 causative gene, we performed whole‐exome sequence analysis in two G2 RCALC1 mice with renal papillary calcification, as well as one WT BALB/c mouse and one WT C3H mouse, in order to determine inheritance of the variants from the parental mice. This analysis identified 20 unique variants, of which three were located on chromosome 17, but only one of these, which involved the bromodomain containing protein 4 (*Brd4*) gene, was within the interval containing the *RCALC1* locus (Supplemental Table S2). This variant was a non‐synonymous heterozygous T‐to‐C transition at nucleotide c.446 in the *Brd4* gene. The T‐to‐C transition in codon 149 of *Brd4* is predicted to cause a missense amino acid change from Met149 to Thr149 (Met149Thr, M149T) (Fig. 2 *B*). Further analysis of the G2 mice (total = 152), by Sanger DNA sequencing (Fig. 2 *B*), revealed that 66% of the mice with renal papillary calcification were heterozygous for the *Brd4* variant (*Brd4^+/M149T^*), and 74% of mice without renal papillary calcification were homozygous for the WT allele (*Brd4^+/+^*) (Table 1). Extension of the co‐segregation analysis to the G3 (*n* = 62), G4 (*n* = 31), and G5 (*n* = 55) mice revealed that 65% of G3, 100% of G4, and 83% of G5 mice with renal papillary calcification were *Brd4^+/M149T^*, consistent with RCALC1 being an autosomal dominant disorder with reduced penetrance and likely due to a BRD4 mutation (Table 1). The effects of the BRD4‐Met149Thr mutation on protein structure were therefore assessed in further detail.

**Table 1:** Co‐segregation of *Brd4^+/M149^* ^*T*^ Variant in RCALC1 Mice

		Renal papillary calcification	
Genotype	Generation	Present	Absent
*Brd4^+/M149T^*	G2	50 (66%)	26 (34%)
	G3	11 (65%)	6 (35%)
	G4	13 (100%)	0 (0%)
	G5	10 (83%)	2 (17%)
*Brd4^+/+^*	G2	20 (26%)	56 (74%)
	G3	8 (18%)	37 (82%)
	G4	5 (28%)	13 (72%)
	G5	3 (7%)	40 (93%)

The co‐segregation of the *Brd4^+/M149T^* variant with renal calcification was investigated in four generations (G2–G5) of the RCALC1 mice. The *Brd4* mutation co‐segregated with the renal papillary calcification phenotype observed in RCALC1 mice over four generations. Between 72% and 93% of *Brd4^+/+^* mice did not have renal papillary calcification.

### Characterization of the BRD4‐Met149Thr variant on protein structure

To assess the potential pathogenicity of the BRD4‐Met149Thr variant, we first examined online databases containing sequencing data from exome analysis in mice and humans. The BRD4‐Met149Thr variant was absent from mouse populations, assessed using The Jackson Laboratory database of exome sequencing data from 175 strains of mice,^49^ and human populations, assessed using the exome aggregation browser (ExAc), which contains genetic information on 60,706 individuals,^50^ thereby indicating that the BRD4 Met149Thr variant is not a common polymorphism. The BRD4 Met149 residue is also highly evolutionarily conserved (Fig. 2 *C*), and bioinformatic analysis using MutationTaster software^37^ predicted the BRD4‐Met149Thr variant to be damaging. We therefore next assessed the effect of the BRD4‐Met149Thr variant on BRD4 protein structure. The BRD4 protein plays a critical role in transcriptional activation by binding to acetylated histone tails using its tandem bromodomains (BD) BD1 and BD2, and regulates transcription by recruiting proteins involved in nuclear remodeling using its extraterminal (ET) domain and its C‐terminal interaction motif (CTM) (Fig. 2 *D*).^51^, ^52^, ^53^, ^54^ The Met149 residue is located in the αC‐helix of BRD4 BD1 (Fig. 2 *E*), and previous studies have shown that Met149 is critical for histone binding and that mutation to Ala149 impairs this binding.^55^ Structural modeling showed that the Met149 residue forms critical backbone contacts with three residues within αC (Fig. 2 *F*) and likely plays a pivotal role in maintaining the structural integrity of BD1. Mutation to Thr149 is predicted to retain these contacts and form an additional polar contact with Asp145 (Fig. 2 *G*), which may reduce the flexibility of BD1. Thus, the BRD4‐Met149Thr variant is likely to affect protein structure, and we therefore assessed the metabolic phenotype of mice with this mutation in further detail.

### Metabolic phenotype analysis of mice with the BRD4‐Met149Thr mutation

Renal calcification is often associated with metabolic abnormalities including hypercalciuria, and we therefore performed biochemical analyses of plasma and urine samples from WT (*Brd4^+/+^*) and heterozygous mutant *Brd4* (*Brd4^+/M149T^*) mice.^1^, ^6^, ^7^, ^56^ *Brd4^+/M149T^* mice were fertile, grew at similar rates as their *Brd4^+/+^* littermates, had similar body weights, and appeared morphologically normal. Analyses of plasma and urine samples from *Brd4^+/+^* and *Brd4^+/M149T^* adult mice, aged 16 weeks, revealed no significant differences between *Brd4^+/+^* and *Brd4^+/M149T^* mice in plasma concentrations of sodium, potassium, albumin‐adjusted calcium, chloride, urea, creatinine, glucose, phosphate, alkaline phosphatase activity, PTH, or 25OHD (Table 2). Furthermore, there were no significant differences between *Brd4^+/+^* and *Brd4^+/M149T^* mice in urine output or pH (Table 3). There were also no significant differences in the urinary excretion of calcium, phosphate, sodium, potassium, magnesium, urate, citrate, or protein, which have previously been associated with renal calcification^57^ (Table 3). An assessment of bone turnover showed no significant differences in plasma P1NP or urine CTx concentrations (Tables 2 and 3). Therefore, RCALC1 is a representative model for idiopathic renal calcification with no associated biochemical abnormality.

**Table 2:** Plasma Biochemical Studies of *Brd4^+/M149^* ^*T*^ Mice

	Male		Female	
	*Brd4^+/+^*	*Brd4^+/M149T^*	*Brd4^+/+^*	*Brd4^+/M149T^*
	*n* = 15	*n* = 5	*n *= 14	*n *= 7
Sodium (mmol/L)	155 ± 3.3	154 ± 1.1	152 ± 2.3	151 ± 2.4
Potassium (mmol/L)	7.26 ± 0.9	7.52 ± 1.5	7.33 ± 0.8	6.91 ± 0.8
Calcium (mmol/L)^a^	2.43 ± 0.2	2.35 ± 0.1	2.46 ± 0.1	2.35 ± 0.1
Chloride (mmol/L)	115 ± 3.3	115 ± 1.5	115 ± 2.5	115 ± 2.0
Urea (mmol/L)	11.4 ± 1.8	11.4 ± 1.7	8.41 ± 2.2	9.65 ± 0.9
Creatinine (μmol/L)	31.9 ± 2.2	30.2 ± 1.9	33.0 ± 2.7	31.4 ± 2.2
Glucose (mmol/L)	7.49 ± 1.6	7.78 ± 1.5	8.62 ± 0.5	9.52 ± 0.7
Phosphate (mmol/L)	2.15 ± 0.5	2.01 ± 0.6	2.40 ± 0.6	1.79 ± 0.4
ALP (U/L)	59.7 ± 15.6	64.2 ± 9.6	101.8 ± 20.5	93.0 ± 14.8
PTH (pg/mL)	52.5 ± 7.8	36.4^b^	20.2 ± 4.4	16.7 ± 6.8
25OHD (ng/mL)	43.0 ± 2.4	32.5^b^	42.5 ± 1.5	40.4 ± 3.0
P1NP (ng/mL)	23.1 ± 1.5	22.1^b^	25.9 ± 2.1	20.2 ± 2.0

ALP = alkaline phosphatase activity; PTH = parathyroid hormone; 25OHD = 25‐hydroxyvitamin D; P1NP = procollagen type 1 N‐terminal propeptide.

Plasma biochemical analysis was performed on 16‐week‐old wild‐type (*Brd4^+/+^*) and RCALC1 (*Brd4^+/M149T^*) mice. All values are expressed as mean ± SD.

^a^ Plasma calcium concentrations were adjusted for the plasma albumin concentration.

^b^ Plasma biochemistry was obtained from 3 male *Brd4^+/M149T^* mice. Mean values are shown in Table 2. All values from male *Brd4^+/M149T^* mice are within 2 SD of the mean values for male WT mice. The individual values from male *Brd4^+/M149T^* mice are as follows: PTH: 34.3, 20.5, and 54.3 pg/mL; 25OHD: 29.5, 30.5, and 37.4 ng/mL; P1NP: 22.2, 25.0, and 19.2 ng/mL.

**Table 3:** Urine Biochemical Studies of *Brd4^+/M149^* ^*T*^ Mice

	Male		Female	
	*Brd4^+/+^*	*Brd4^+/M149T^*	*Brd4^+/+^*	*Brd4^+/M149T^*
	*n* = 10–15	*n* = 4	*n* = 10–14	*n* = 5–7
Urine output (mL/24 h)	2.35 ± 1.03	2.63 ± 1.98	2.16 ± 2.03	1.84 ± 0.53
pH	6.96 ± 0.60	7.07 ± 0.42	6.74 ± 0.77	6.37 ± 0.24
Calcium (μmol/24 h)	0.67 ± 0.41	0.58 ± 0.59	1.64 ± 0.65	1.22 ± 0.33
Phosphate (mmol/24 h)	0.06 ± 0.03	0.10 ± 0.02	0.09 ± 0.02	0.09 ± 0.02
Magnesium (μmol/24 h)	19.3 ± 7.8	11.5 ± 11.2	31.9 ± 18.4	39 ± 8.9
Urate (μmol/24 h)	0.51 ± 0.14	0.48 ± 0.12	0.48 ± 0.14	0.73 ± 0.43
Citrate (nmol/24 h)	5.2 ± 3.3	3.0 ± 1.4	2.6 ± 0.6	2.7 ± 0.9
Sodium (mmol/24 h)	0.32 ± 0.05	0.28 ± 0.04	0.41 ± 0.07	0.44 ± 0.08
Potassium (mmol/24 h)	0.58 ± 0.09	0.47 ± 0.06	0.75 ± 0.10	0.80 ± 0.10
CTx (μg/24 h)	0.21 ± 0.08	0.16 ± 0.08	0.68 ± 0.20	0.48 ± 0.20
Creatinine (μmol/24 h)	7.3 ± 1.3	6.4 ± 1.0	10.0 ± 0.9	10.4 ± 2.8
Protein (mg/dL)	735.18 ± 247.11	884.36 ± 389.22	341.40 ± 179.68	395.3 ± 158.1

Urine biochemical analysis was performed on 16‐week‐old wild‐type (*Brd4^+/+^*) and RCALC1 (*Brd4^+/M149T^*) mice, in metabolic cages, using urine samples collected over a 24‐hour period. All values are expressed as mean ± SD.

### Effect of the BRD4*‐*Met149Thr mutation on gene transcription

**Figure 3:**
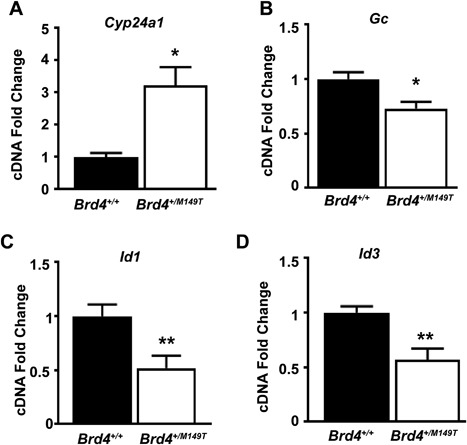
Analysis of differentially expressed genes by qRT‐PCR. (*A–D*) Validation of differentially expressed genes from cDNA expression profiling analysis of kidneys from wild‐type (*Brd4^+/+^*) and RCALC1 (*Brd4^+/M149T^*) mice by qRT‐PCR analysis. Renal expression of (*A*) *Cyp24a1*, (*B*) *Gc*, (*C*) *Id1*, and (D) *Id3* in cDNA from *Brd4^+/+^* and *Brd4^+/M149T^* mice. *n* = 4–6 mice per group. Data were normalized to geometric mean of three housekeeper genes (*Ccnd1*, *Ccnd2*, *Gapdh*) and expressed as a fold‐change relative to *Brd4^+/+^*, set at 1. Histograms are presented as mean ± SEM. Statistical analyses were performed by Student's *t* test, *p < 0.05, **p < 0.02, compared with *Brd4^+/+^*.

BRD4 plays a critical role in histone modification and gene transcription, and previous studies have shown that the Met149 residue is important for histone binding.^55^ We therefore hypothesized that the *Brd4^+/M149T^* mutation may lead to changes in gene transcription, which may explain the renal calcification phenotype observed in RCALC1 mice. To assess gene transcription, we performed cDNA expression profiling using whole kidneys from four *Brd4^+/M149T^* and eight *Brd4^+/+^* mice from the parental strains (4 BALB/c mice and 4 C3H mice). We used kidneys because it was not possible to use microdissected specimens of calcified regions from the papilla, as these calcified regions had a mean diameter of 30.56 µm (range 8 to 70 µm) (sections from *n* = 63 mice), and the mean area of calcification was 725.83 µm^2^ (range 50.24 to 3846.5 µm^2^) and therefore too small to extract high‐quality RNA that would allow for the cDNA expression profiling studies. These analyses of kidneys revealed 37 genes to be significantly differentially expressed by at least 1.50‐fold in *Brd4^+/M149T^* mice, when compared with *Brd4^+/+^* mice (Supplemental Tables S3 and S4). Eighteen genes were significantly upregulated and 19 genes significantly downregulated in *Brd4^+/M149T^* kidneys when compared with *Brd4^+/+^* kidneys (Table S3 and S4). This included two genes involved in regulating vitamin D_3_ and calcium homeostasis. Thus, the vitamin D_3_‐24‐hydroxylase transcript cytochrome P450, family 24, subfamily A, polypeptide 1 (*Cyp24a1*), which encodes the 24‐hydroxylase enzyme that degrades active 1,25‐dihydroxyvitamin D_3_ to inactive vitamin D metabolites, was 5.05‐fold higher in *Brd4^+/M149T^* kidneys compared with *Brd4^+/+^* kidneys; whereas expression of the vitamin D binding protein (*Gc*, group‐specific component) was 1.55‐fold lower in *Brd4^+/M149T^* kidneys compared with *Brd4^+/+^* kidneys. Furthermore, qRT‐PCR in kidneys from 4 *Brd4^+/+^* and 4 *Brd4^+/M149T^* mice confirmed these results (Fig. 3 *A*, *B*), thereby supporting the role of disturbed vitamin D metabolism in the etiology of the renal calcification observed in RCALC1 mice.

**Figure 4:**
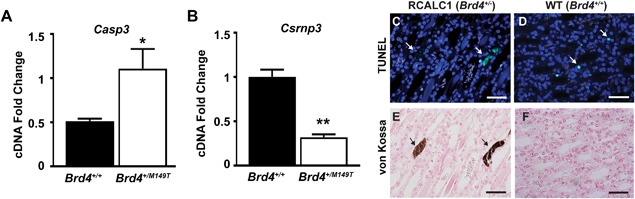
Analysis of apoptosis in *Brd4^+/M149T^* mice. (*A*, *B*) Validation of differentially expressed genes involved in apoptosis from cDNA expression profiling analysis of kidneys from wild‐type (*Brd4^+/+^*) and RCALC1 (*Brd4^+/M149T^*) mice by qRT‐PCR analysis. Renal expression of (*A*) *Casp3* and (*B*) *Csrnp3* genes, in kidney cDNA from *Brd4^+/+^* and *Brd4^+/M149T^* mice. *n* = 4–6 mice for each group. Data were normalized to the geometric mean of three housekeeper genes (*Ccnd1*, *Ccnd2*, *Gapdh*) and expressed as a fold‐change relative to *Brd4^+/+^*, set at 1. Histograms are presented as mean ± SEM. Statistical analyses were performed by Student's *t* test, *p < 0.05, **p < 0.02, compared with *Brd4^+/+^*. (*C*, *D*) TUNEL and (*E*, *F*) von Kossa staining showing apoptotic nuclei (green) in areas corresponding to interstitial calcification in RCALC1 (*Brd4^+/M149T^*) mice (arrows) and in control wild‐type (WT) *Brd4^+/+^* mice. Nuclei were counterstained with nuclear fast red for von Kossa staining and DAPI (blue) for immunofluorescence. Scale bars = 50 μm.

A number of genes involved in cell survival and differentiation were also found to be differentially expressed in *Brd4^+/M149T^* kidneys, when compared with *Brd4^+/+^* kidneys (Supplemental Tables S3 and S4). The inhibitor of DNA binding 1 (*Id1*) and 3 (*Id3*) genes, which play a role in cell differentiation, were downregulated in *Brd4^+/M149T^* kidneys compared with *Brd4^+/+^* (–3.91‐fold for *Id1* and –3.24*‐*fold for *Id3*) (Supplemental Tables S3 and S4, Fig. 3 *C*, *D*). In addition, genes involved in apoptosis were also differentially expressed. The gene encoding the pro‐apoptosis protein caspase 3 (*Casp3*) was upregulated by 1.64‐fold, whereas the cysteine‐serine‐rich nuclear protein 3 (*Csrnp3*), which is thought to have a role in transforming growth factor (TGF)‐β‐induced apoptosis,^58^ was downregulated by 1.60‐fold in *Brd4^+/M149T^* kidneys compared with *Brd4^+/+^* kidneys (Supplemental Tables S3 and S4, Fig. 4 *A*, *B*). Previous studies of cultured renal cells and rat models of NL have suggested that apoptosis promotes adhesion and retention of calcium containing crystals,^59^, ^60^, ^61^ and because we had observed changes in expression of genes that are known to be important in apoptosis, we further sought to determine whether increased apoptosis was present in *Brd4^+/M149T^* kidneys. Apoptosis was assessed in serial sections from kidneys of *Brd4^+/M149T^* and *Brd4^+/+^* mice using TUNEL staining and von Kossa staining (Fig. 4 *C–F*). The percentage of apoptotic nuclei in the renal papillae of *Brd4^+/M149T^* mice (*n* = 7) was not significantly different from that observed in *Brd4^+/+^* mice (*n* = 4) (1.13% ± 0.33% versus 3.36% ± 1.24%, respectively, *t* test *p* > 0.1). However, in 3 of the 7 kidneys from *Brd4^+/M149T^* mice examined, TUNEL‐labeling was observed in interstitial regions of the renal papilla corresponding to sites of calcification (Fig. 4 *C*, *E*), consistent with apoptosis of interstitial cells, which may comprise renal fibroblasts and immune cells.^62^ Such labeling was absent in the renal papillae of WT (*Brd4^+/+^*) mice (Fig. 4 *D*, *F*). Therefore, *Brd4^+/M149T^* mice likely have increased apoptosis at sites of calcification within the renal papilla.

## Discussion

Our studies have established a mouse model for an autosomal dominant form of renal calcification, designated RCALC1, which is due to a heterogeneous germline Met149Thr mutation of *Brd4* and has phenotypic similarities to those observed in patients with NC.^4^ Thus, RCALC1 mutant mice (*Brd4^+/M149T^*) had the interstitial renal papillary calcium deposits that were similar to the papillary calcification that accompanies Randall's plaque, a lesion observed in 85% to 95% of humans with calcium phosphate (apatite) and calcium oxalate deposits.^4^, ^5^, ^46^ Thus, in humans, Randall's plaque has been reported to contain interstitial apatite deposits that act as a nidus for urothelial surface deposition of calcium oxalate,^4^, ^45^ although calcium oxalate has been reported to overlie and adhere to Randall's plaque in only 50% of stone formers.^47^ In addition, previous studies of plaque formation in patients with recurrent stones have demonstrated that most stones grow by remaining attached to papillae and that subsequent crystal deposition involves the stone‐tissue interface becoming overlaid with osteopontin and THP.^4^, ^44^, ^46^ This is consistent with observations that osteopontin is involved in the formation of organic layers of the plaques^45^ and that molecules from the urine, such as THP, attach to these sites and extend the stone into the urinary space.^44^ Our findings that the RCALC1 (*Brd4^+/M149T^*) mice have calcification, detected by von Kossa staining, at the interstitial renal papilla that is co‐localized with osteopontin and THP (Fig. 1) indicate that renal calcification in the *Brd4^+/M149T^* mice may involve a Randall's plaque‐like origin, similar to that observed in humans with NC.^4^, ^45^ Thus, our findings suggest that the RCALC1 mutant mouse may represent a model for NC. Moreover, the RCALC1 mutant mice have similarities with other mouse models of NC, which also have interstitial papillary calcification, and these include mice null for THP,^63^, ^64^ osteopontin,^63^ and the sodium‐hydrogen exchanger regulator factor 1 (NHERF1).^65^ Furthermore, the absence of plasma and urinary biochemical abnormalities in the RCALC1 mice is also similar to that reported in osteopontin‐null mice,^63^ whereas the lack of calcium oxalate deposits at the sites of papillary calcification in the RCALC1 mice is similar to that reported in the THP,^63^, ^64^ osteopontin,^63^ and NHERF1^65^ knockout mice. Thus, the RCALC1 mice, which have a *Brd4* mutation, provide another model for studying the process of calcification in Randall's plaques that is observed in patients with NC.^4^, ^45^

Our results, showing that a *Brd4* mutation (Met149Thr) is associated with renal calcification in the RCALC1 mice, also reveal a new pathogenic role for the encoded protein, BRD4, which plays a critical role in regulating gene transcription by binding to modified histone tails of chromosomal DNA and recruiting transcriptional activating proteins.^(51–54)^ Our structural studies demonstrated that mutation of Met149 to Thr149 is likely to affect protein structure (Fig. 2), and previous studies have shown the Met149 residue to be required for histone binding.^55^ Furthermore, Met149 is highly conserved (Fig. 2), and the Thr149 mutation of this residue has not previously been identified in exome data sets from humans or mice and co‐segregates with the renal calcification phenotype. It is therefore likely that the mutation is pathogenic and is causative for the renal calcification in RCALC1 mice. Moreover, our cDNA expression profiling results showed that the BRD4*‐*Met149Thr mutation resulted in altered expression of *Cyp24a1* and *Gc*, which are key genes in vitamin D metabolism and whose dysregulation in the kidneys (Fig. 3) may result in intracellular effects that cause renal calcification without altering systemic calcium homeostasis (Tables 2 and 3), as observed to occur in some patients with NC.^4^ Such underlying mechanisms that may cause renal calcification, without altering systemic calcium homeostasis, include cell survival, differentiation, and apoptosis, and our cDNA expression profiling studies revealed decreased expression of *Id1* and *Id3* (Fig. 3 *C*, *D*), which have roles in cell differentiation, and altered expression of *Casp3* and *Csrnp3* (Fig. 4 *A*, *B*), which have roles in apoptosis. Indeed, our studies of RCALC1 mice demonstrated that kidneys from the *Brd4^+/M149T^* mice had apoptosis of renal papillary interstitial cells in association with the calcified lesions (Fig. 4 *C*, *D*). These results are consistent with those from other studies that have reported an increased apoptosis and differential expression of genes involved in proliferation in Randall's plaques from stone formers when compared with a control group of patients.^48^ Furthermore, stone formation in rats is reported to be associated with an increase in apoptosis, and studies in renal cells have also shown that calcium phosphate stones are associated with apoptosis and changes in cell proliferation.^48^, ^66^, ^67^ Thus, renal injury and cell death may be common mechanisms in the etiology of renal calcification and the resulting NC and NL by generating sites that promote calcium crystal aggregation and growth.^59^, ^68^

On the basis of these findings, we propose the following possible model for the interstitial renal papillary calcification and likely NC in RCALC1 mice. In the RCALC1 *Brd4^+/M149T­^* mice, the BRD4‐Met149Thr mutation will impair gene transcription by affecting histone binding, which results in aberrant expression of genes encoding proteins involved in cell survival and differentiation that are known to be reliant on histone modification.^53^ Failure of these pathways leads to increased apoptosis in renal papillary cells and promotes tissue damage, resulting in crystal retention and NC. In addition, dysregulation of the vitamin D metabolism pathway may result in intracellular toxic effects that exacerbate apoptosis.^69^ In summary, the RCALC1 mouse, which is associated with a BRD4 missense mutation, represents a model of idiopathic renal calcification that provides an in vivo resource for mechanistic studies of NC.

## Disclosures

RVT received grant funding from NPS/Shire Pharmaceuticals, GlaxoSmithKline, Novartis Pharma AG, and the Marshall Smith Syndrome Foundation for unrelated studies. All other authors state that they have no conflicts of interest.

## Supporting information

Supporting Data S1.
