# Supplementary material for: Mice with a Brd4 Mutation Represent a New Model of Nephrocalcinosis
Source: J Bone Miner Res. 2019 Mar 4;34(7):1324–35. doi: 10.1002/jbmr.3695 (PMC6658219; doi:10.1002/jbmr.3695)
Supplement: Supplementary file 1 — Supporting Data S1. [file JBMR-34-1324-s001.pdf]

Table S1 Genes associated with nephrocalcinosis or nephrolithiasis in humans identified in association studies or as part of a syndrome

Disease/ association	Gene	Gene Name	Reference
Adenine phosphoribosyltransferase deficiency	*APRT*	Adenine phosphoribosyltransferase	(1)
Associated with calcium NL	*SLC26A1*	Solute Carrier family 26 (sulfate transporter), Member 1	(2)
Associated with calcium NL	*SLC26A6*	Solute Carrier family 26 (anion exchanger), Member 6	(3)
Associated with kidney stones	*ALPL*	Alkaline phosphatase, liver/ kidney/ bone	(1)
Associated with kidney stones	*CLDN14*	Claudin 14	(1,4,5)
Associated with NL	*ITPKC*	Inositol 1,4,5-trisphosphate (IP3) 3-kinase C	(2)
Associated with NL	*ORAI1*	Orai calcium release-activated calcium modulator 1	(6)
Associated with NL	*SSP1*	Secreted phosphoprotein 1, osteopontin	(7)
Associated with NL	*TRPV5*	Transient receptor potential cation channel subfamily V, member 5	(1,8,9)
Associated with NL	*UMOD*	Uromodulin	(10)
Autosomal dominant hypocalcaemia; Bartter syndrome type 5	*CASR*	Calcium-sensing receptor	(1,11-14)
Bartter syndrome type 1; association with NC/NL	*SLC12A1*	Solute carrier family 12, member 1	(2,12,15-17)
Bartter syndrome type 2	*ROMK*	Renal outer medullary potassium channel	(18)
Bartter syndrome type 2	*KCNJ1*	Potassium inwardly-rectifying channel, subfamily J, member 1	(19)
Bartter syndrome type 3	*CLCNKB*	Chloride channel Kb	(4,11)
Bartter syndrome type 4	*BSND*	Barttin	(20)
Cystinuria type A; association with NC/NL	*SLC3A1*	Solute carrier family 3 (cystine, dibasic and neutral amino acid transporters), member 1	(21-23)
Cystinuria type B	*SLC7A9*	Solute carrier family 7 (glycoprotein associated amino acid transporter light chain), member 9	(24)
Distal renal tubular acidosis (dRTA); association with NC/NL	*ATP6V0A4*	ATPase, H^+ transporting, lysosomal V0 subunit a4	(25-27)
dRTA; association with NC/NL	*SLC4A1*	Solute carrier family 4, anion exchanger, member 1 (erythrocyte membrane protein band 3)	(26-28)
dRTA with deafness; association with NC/NL	*ATP6V1B1*	ATPase, H^+ transporting, lysosomal 56/58kDa, V1 subunit B1	(12,24,29-31)
Dent's disease type 1	*CLCN5*	Chloride channel 5	(4,11,32)
Enamel-Renal syndrome, amelogenesis imperfect and NC	*FAM20A*	Family with sequence similarity 20, member A	(33)
Familial hypomagnesemia with hypercalciuria and NC (FHHNC); association with NC/NL	*CLDN16*	Claudin 16	(5,12,34,35)
FHHNC with ocular abnormalities; association with NC/NL	*CLDN19*	Claudin 19	(2,12)
Hypophosphatasia; association with NL	*ALPL*	Alkaline phosphatase, Liver/Bone/Kidney	(1,36)
Hypophosphatemic NL/ osteoporosis-1/ Fanconi renotubular syndrome 2; Idiopathic infantile hypercalcemia; association with NC/NL	*SLC34A1*	Solute carrier family 34 (sodium phosphate), member 1	(1,2,12,37,38)
Hypophosphatemic NL/osteoporosis-2; association with NC/NL	*SLC9A3R1*	Solute carrier family 9, subfamily A (cation proton antiporter 3), member 3 regulator 1	(12,29,39,40)
Hypophosphatemic rickets with hypercalciuria	*SLC34A3*	Solute carrier family 34 (sodium phosphate), member 3	(2,3)
Idiopathic (absorptive) hypercalciuria, susceptibility; association with NC/NL	*ADCY10/SAC*	Adenylate cyclase 10 (soluble)	(1,29)
Infantile hypercalcemia; association with NC/NL	*CYP24A1*	Cytochrome P450, family 24, subfamily A, polypeptide 1	(33,41)
Idiopathic hypercalciuria; association with NC/NL	*VDR*	Vitamin D (1,25- dihydroxyvitamin D3) receptor	(40,42,43)
Kelley-Seegmiller syndrome, partial HPRT deficiency, HPRT-related gout	*HPRT1*	Hypoxanthine phosphoribosyltransferase 1	(44)
Lowe syndrome / Dent disease 2; association with NC/NL	*OCRL*	Oculocerebrorenal syndrome of Lowe	(19,45)
Maturity-onset diabetes of the young (MODY) with Fanconi syndrome and NC	*HNF4A*	Hepatocyte nuclear factor 4, alpha	(41)
Nephropathic cystinosis	*CTNS*	Cystinosin, lysosomal cystine transporter	(46)

Osteopetrosis with dRTA	*CA2*	Carbonic anhydrase II	[1,11-14,47]
Primary hyperoxaluria, type 1; association with NC/NL	*AGXT*	Alanine-glyoxylate aminotransferase	[24,26,29,48]
Primary hyperoxaluria type 2	*GRHPR*	Glyoxylate reductase/hydroxypyruvate reductase	[41]
Primary hyperoxaluria type 3	*HOGA1*	4-hydroxy-2-oxoglutarate aldolase 1	[2]
Renal hypouricemia type 1	*SLC22A12*	Solute carrier family 22 (organic anion/urate transporter), member 12	[2]
Renal hypouricemia type 2	*SLC2A9*	Solute carrier family 2 (facilitated glucose transporter), member 9	[28]
Xanthinuria type 1	*XDH*	Xanthine dehydrogenase	[49]

NC, nephrocalcinosis; NL, nephrolithiasis.

Table S2 Variants found in RCALC1 mice by exome capture

Gene	Gene name	Nucleotide change	Amino acid change	Mutation type	Chromosome
Gigyf2	GRB10 interacting GYF protein 2	c.495-2A>G	N/A	Splice site	1
Pappa2	pappalysin 2	c.T2686C	p.Y896H	Nonsynonymous SNV	1
Mcoln2	mucolipin 2	c.G172T	p.A58S	Nonsynonymous SNV	3
Mast2	microtubule associated serine/threonine kinase 2	c.T651A	p.F217L	Nonsynonymous SNV	4
Tekt2	tektin 2	c.T131C	p.I44T	Nonsynonymous SNV	4
Copg	coatomer protein complex, subunit gamma	c.171+4A>G	N/A	Splice site	6
Pik3c2g	phosphatidylinositol 3-kinase, C2 domain containing, gamma polypeptide	c.G1861T	p.V621L	Nonsynonymous SNV	6
Gltscr1	glioma tumor suppressor candidate region gene 1	c.3216+4A>G	N/A	Splice site	7
Oca2	oculocutaneous albinism II	c.T2414C	p.F805S	Nonsynonymous SNV	7
Cenpn	centromere protein N	c.G931A	p.G311R	Nonsynonymous SNV	8
Cdk4	cyclin-dependent kinase 4	c.G365A	p.R122H	Nonsynonymous SNV	10
Med13	mediator complex subunit 13	c.C4598T	p.T1533I	Nonsynonymous SNV	11
Zpbp2	zona pellucida binding protein 2	c.A685G	p.T229A	Nonsynonymous SNV	11
Prpf4b	PRP4 pre-mRNA processing factor 4 homolog B	c.T2750C	p.V917A	Nonsynonymous SNV	13
Pabpc1	poly(A) binding protein, cytoplasmic 1	c.T1018G	p.F340V	Nonsynonymous SNV	15
Chd1	chromodomain helicase DNA binding protein 1	c.3978+2T>C	N/A	Splice site	17
Brd4	bromodomain containing 4	c.T446C	p.M149T	Nonsynonymous SNV	17
Fshr	follicle stimulating hormone receptor	c.A218G	p.E73G	Nonsynonymous SNV	17
Matr3	matrin 3	c.T2046A	p.N682K	Nonsynonymous SNV	18
Xpnpep1	X-prolyl aminopeptidase (aminopeptidase P) 1, soluble	c.1003-3C>A	N/A	Splice site	19

Unique single nucleotide variants (SNV) found in the DNA of two G2 RCALC1 mice with renal papillary calcification, compared to Balb/c and C3H parental strains. Three genes are located on chromosome 17, and only one of these, the *Brd4* gene, is situated within the interval between *rs33662699* and *D17mit51* that encompasses the *RCALC1* locus (Fig. 2). N/A, not applicable for splice site mutations.

Table S3 Upregulated genes in kidneys of *Brd4*$^{+/M149T}$ (RCALC1) mice compared to parental Balb/c and C3H mice (*Brd4*$^{+/+}$)

Gene	Gene name	Fold-change to Balb/c	Fold-change to C3H
Ube2c	ubiquitin-conjugating enzyme E2C	+16.49**	+1.76**
Serpina1a	serine (or cysteine) peptidase inhibitor, clade A, member 1A	+14.60**	+2.05**
Angptl4	angiopoietin-like 4	+4.08**	+2.06**
Cyp24a1	cytochrome P450, family 24, subfamily a, polypeptide 1	+5.05**	+2.83**
Cyp2e1	cytochrome P450, family 2, subfamily e, polypeptide 1	+3.57**	+1.68**
Hist2h3c1	histone cluster 2, H3c1	+3.26**	+1.63*
Fxyd2	FXYD domain-containing ion transport regulator 2	+2.23**	+2.43**
Pdk4	pyruvate dehydrogenase kinase, isoenzyme 4	+2.20**	+3.20**
Ppara	peroxisome proliferator activated receptor alpha	+2.31**	+1.52**
Acox1	acyl-coenzyme A oxidase 1, palmitoyl	+2.06**	+1.81**
Mt2	metallothionein 2	+2.05**	+1.57**
Slc25a42	solute carrier family 25, member 42	+1.99**	+1.52**
Susd3	sushi domain containing 3	+1.98**	+1.59**
Nkain1	Na+/K+ transporting ATPase interacting 1	+1.79**	+1.74**
Sypl2	synaptophysin-like 2	+1.73**	+1.63**
Ehf	ets homologous factor	+1.69**	+1.52**
Casp3	caspase 3	+1.64**	+1.66**
Pxmp4	peroxisomal membrane protein 4	+1.55**	+1.59**

Genes upregulated in kidneys of *Brd4*$^{+/M149T}$ mice compared to parental Balb/c and C3H mice (wild-type (*Brd4*$^{+/+}$)). Pair-wise comparisons of expression data were performed using the least significant difference (LSD) test. **p<0.02, *p<0.05.

Table S4 Downregulated genes in kidneys of *Brd4*$^{+/M149T}$ (RCALC1) mice compared to parental Balb/c and C3H mice (*Brd4*$^{+/+}$)

Gene	Gene name	Fold-change to Balb/c	Fold-change to C3H
Gabrb3	gamma-aminobutyric acid (GABA) A receptor, subunit beta 3	-16.57**	-1.73**
Id1	inhibitor of DNA binding 1	-3.91**	-2.01**
Mdk	Midkine	-3.87**	-1.95**
Slc16a14	solute carrier family 16 (monocarboxylic acid transporters), member 14	-3.57**	-3.46**
Guca2a	guanylate cyclase activator 2a	-3.40**	-1.77**
Id3	inhibitor of DNA binding 3	-3.24**	-1.98**
Fabp4	fatty acid binding protein 4, adipocyte	-2.75**	-1.70**
Asb9	ankyrin repeat and SOCS box-containing 9	-2.54**	-1.82**
Abcc4	ATP-binding cassette, sub-family C, member 4	-2.49**	-1.75**
Sash1	SAM and SH3 domain containing 1	-2.28**	-1.62**
Btnl9	butyrophilin-like 9	-2.18**	-1.57**
Rbm12	RNA binding motif protein 12	-2.11**	-1.67**
Klf4	Kruppel-like factor 4	-2.11**	-1.64**
Ablim3	actin binding LIM protein family, member 3	-1.66**	-1.79**
Pla2g7	phospholipase A2, group VII	-1.65**	-2.04**
Csrnp3	cysteine-serine-rich nuclear protein 3	-1.60**	-1.87**
Gc	group specific component	-1.55**	-1.57**
Ets1	E26 avian leukemia oncogene 1, 5' domain	-1.53**	-1.54**
Arvcf	armadillo repeat gene deleted in velo-cardio-facial syndrome	-1.51**	-1.82**

Genes downregulated in kidneys of *Brd4*$^{+/M149T}$ mice compared to parental Balb/c and C3H mice (wild-type (*Brd4*$^{+/+}$)). Pair-wise comparisons of expression data were performed using the least significant difference (LSD) test. *$p<0.05$, **$p<0.02$.

Fig. S1 Examination of RCALC1 kidneys for the presence of calcium oxalate

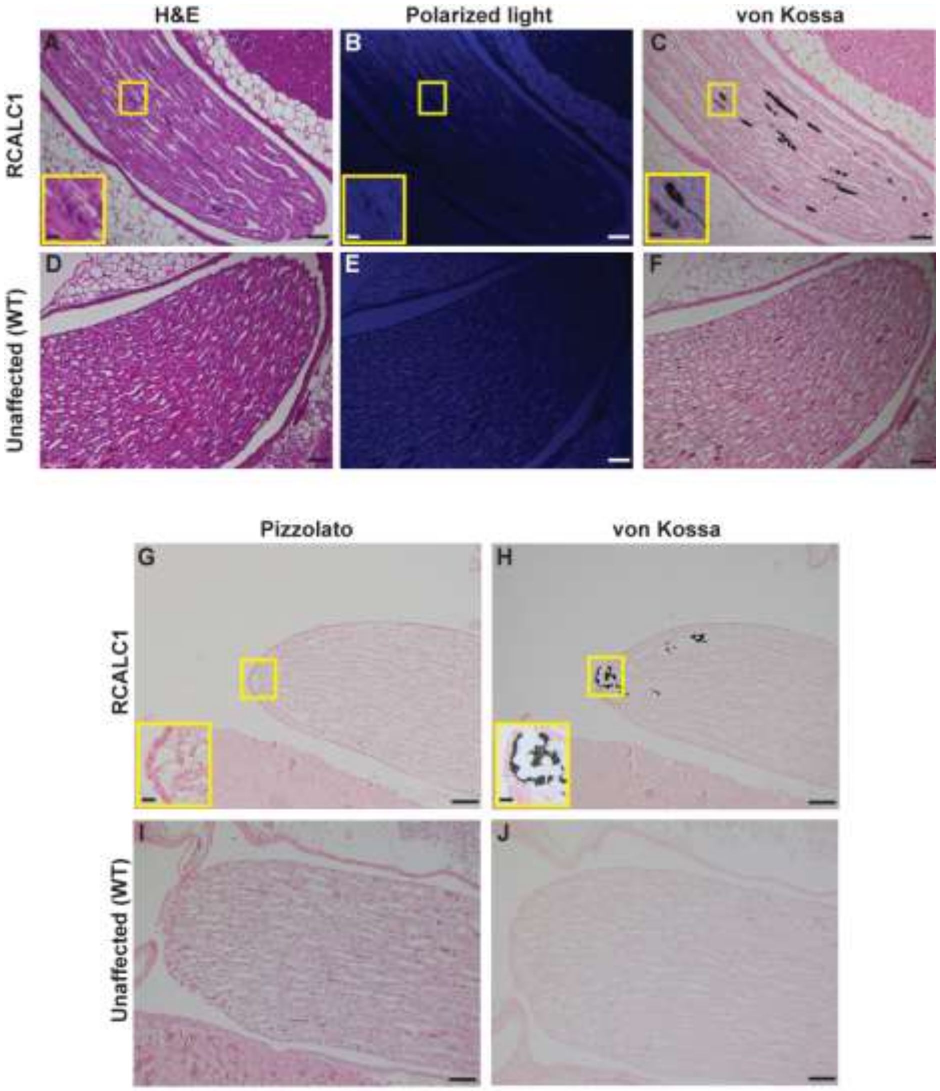

Assessment of renal calcification for calcium oxalate by polarized light microscopy and Pizzolato staining, in RCALC1 mice (n=12, 6 males and 6 females) and unaffected wild-type (WT) control littermates (n=12, 6 males and 6 females). (**A**-**C**) Examination of renal sections from a RCALC1 mouse by (**A**) haematoxylin and eosin (H&E) and (**B**) polarized light, and (**C**) of a serial section by von Kossa staining. (**D**-**F**) Examination of a renal section from an unaffected wild-type (WT) control littermate by (**D**) H&E and (**E**) polarized light, and (**F**) of a serial section by Von Kossa staining. (**G**-**H**) Examination of

renal serial sections from a RCALC1 mouse by (**G**) Pizzolato and (**H**) von Kossa staining. (**I-J**) Examination of renal serial sections from unaffected WT control littermates by (**I**) Pizzolato and (**J**) von Kossa staining. H&E sections from the RCALC1 mouse had blue-purple interstitial inclusions (A and inset), which were not birefringent under polarized light (B and inset) but appeared as brown-black deposits with von Kossa stains (C and inset), indicating that these crystals are not formed of calcium oxalate but are formed of calcium phosphate, respectively. Pizzolato staining of RCALC1 renal serial sections did not reveal the brown-black staining associated with calcium-oxalate crystals (G and inset), but did reveal brown-black staining with von Kossa staining (H and inset), thereby confirming that the interstitial deposits do not consist of calcium-oxalate, but instead consist of calcium phosphate. Renal sections from unaffected WT littermates did not contain interstitial inclusions (D), calcium oxalate (E and I) crystals, or calcium phosphate deposits (F and J). Scale bars: 100µm (main panel) and 10µm (inset).

References

1. Oddsson A, Sulem P, Helgason H, Edvardsson VO, Thorleifsson G, Sveinbjornsson G, et al. Common and rare variants associated with kidney stones and biochemical traits. Nat Commun. 2015;6:7975.
2. Loh NY, Bentley L, Dimke H, Verkaart S, Tammaro P, Gorvin CM, et al. Autosomal dominant hypercalciuria in a mouse model due to a mutation of the epithelial calcium channel, TRPV5. PloS one. 2013;8(1):e55412.
3. Gorvin CM, Wilmer MJ, Piret SE, Harding B, van den Heuvel LP, Wrong O, et al. Receptor-mediated endocytosis and endosomal acidification is impaired in proximal tubule epithelial cells of Dent disease patients. Proceedings of the National Academy of Sciences of the United States of America. 2013;110(17):7014-9.
4. Thorleifsson G, Holm H, Edvardsson V, Walters GB, Styrkarsdottir U, Gudbjartsson DF, et al. Sequence variants in the CLDN14 gene associate with kidney stones and bone mineral density. Nat Genet. 2009;41(8):926-30.
5. Piret SE, Thakker RV. Mouse models for inherited endocrine and metabolic disorders. The Journal of endocrinology. 2011;211(3):211-30.
6. Wang K, Li M, Hakonarson H. ANNOVAR: functional annotation of genetic variants from high-throughput sequencing data. Nucleic acids research. 2010;38(16):e164.
7. Longley MJ, Clark S, Yu Wai Man C, Hudson G, Durham SE, Taylor RW, et al. Mutant POLG2 disrupts DNA polymerase gamma subunits and causes progressive external ophthalmoplegia. American journal of human genetics. 2006;78(6):1026-34.
8. Virgilio R, Ronchi D, Hadjigeorgiou GM, Bordoni A, Saladino F, Moggio M, et al. Novel Twinkle (PEO1) gene mutations in mendelian progressive external ophthalmoplegia. Journal of neurology. 2008;255(9):1384-91.
9. Galmiche L, Serre V, Beinat M, Assouline Z, Lebre AS, Chretien D, et al. Exome sequencing identifies MRPL3 mutation in mitochondrial cardiomyopathy. Human mutation. 2011;32(11):1225-31.
10. Vervaet BA, Verhulst A, D'Haese PC, De Broe ME. Nephrocalcinosis: new insights into mechanisms and consequences. Nephrology, dialysis, transplantation : official publication of the European Dialysis and Transplant Association - European Renal Association. 2009;24(7):2030-5.
11. Braun DA, Lawson JA, Gee HY, Halbritter J, Shril S, Tan W, et al. Prevalence of Monogenic Causes in Pediatric Patients with Nephrolithiasis or Nephrocalcinosis. Clinical journal of the American Society of Nephrology : CJASN. 2016;11(4):664-72.
12. Daga A, Majmundar AJ, Braun DA, Gee HY, Lawson JA, Shril S, et al. Whole exome sequencing frequently detects a monogenic cause in early onset nephrolithiasis and nephrocalcinosis. Kidney international. 2018;93(1):204-13.
13. Shakhssalim N, Kazemi B, Basiri A, Houshmand M, Pakmanesh H, Golestan B, et al. Association between calcium-sensing receptor gene polymorphisms and recurrent calcium kidney stone disease: a comprehensive gene analysis. Scandinavian journal of urology and nephrology. 2010;44(6):406-12.

14. Mohebbi N, Ferraro PM, Gambaro G, Unwin R. Tubular and genetic disorders associated with kidney stones. Urolithiasis. 2017;45(1):127-37.
15. Keane TM, Goodstadt L, Danecek P, White MA, Wong K, Yalcin B, et al. Mouse genomic variation and its effect on phenotypes and gene regulation. Nature. 2011;477(7364):289-94.
16. DePristo MA, Banks E, Poplin R, Garimella KV, Maguire JR, Hartl C, et al. A framework for variation discovery and genotyping using next-generation DNA sequencing data. Nat Genet. 2011;43(5):491-8.
17. Newey PJ, Gorvin CM, Cleland SJ, Willberg CB, Bridge M, Azharuddin M, et al. Mutant prolactin receptor and familial hyperprolactinemia. The New England journal of medicine. 2013;369(21):2012-20.
18. Adzhubei IA, Schmidt S, Peshkin L, Ramensky VE, Gerasimova A, Bork P, et al. A method and server for predicting damaging missense mutations. Nature methods. 2010;7(4):248-9.
19. Li H, Homer N. A survey of sequence alignment algorithms for next-generation sequencing. Briefings in bioinformatics. 2010;11(5):473-83.
20. Hunter DJ, Lange M, Snieder H, MacGregor AJ, Swaminathan R, Thakker RV, et al. Genetic contribution to renal function and electrolyte balance: a twin study. Clin Sci (Lond). 2002;103(3):259-65.
21. Reed AA, Loh NY, Terryn S, Lippiat JD, Partridge C, Galvanovskis J, et al. CLC-5 and KIF3B interact to facilitate CLC-5 plasma membrane expression, endocytosis, and microtubular transport: relevance to pathophysiology of Dent's disease. American journal of physiology Renal physiology. 2010;298(2):F365-80.
22. Martin SA, Hewish M, Sims D, Lord CJ, Ashworth A. Parallel high-throughput RNA interference screens identify PINK1 as a potential therapeutic target for the treatment of DNA mismatch repair-deficient cancers. Cancer research. 2011;71(5):1836-48.
23. Lines KE, Stevenson M, Filippakopoulos P, Muller S, Lockstone HE, Wright B, et al. Epigenetic pathway inhibitors represent potential drugs for treating pancreatic and bronchial neuroendocrine tumors. Oncogenesis. 2017;6(5):e332.
24. Sayer JA, Carr G, Simmons NL. Nephrocalcinosis: molecular insights into calcium precipitation within the kidney. Clinical science. 2004;106(6):549-61.
25. Moochhala SH, Sayer JA, Carr G, Simmons NL. Renal calcium stones: insights from the control of bone mineralization. Exp Physiol. 2008;93(1):43-9.
26. Coe FL, Evan A, Worcester E. Kidney stone disease. J Clin Invest. 2005;115(10):2598-608.
27. Frick KK, Bushinsky DA. Molecular mechanisms of primary hypercalciuria. J Am Soc Nephrol. 2003;14(4):1082-95.
28. Schwarz JM, Cooper DN, Schuelke M, Seelow D. MutationTaster2: mutation prediction for the deep-sequencing age. Nature methods. 2014;11(4):361-2.
29. Scheinman SJ. Nephrolithiasis. Semin Nephrol. 1999;19(4):381-8.
30. Polito C, La Manna A, Nappi B, Villani J, Di Toro R. Idiopathic hypercalciuria and hyperuricosuria: family prevalence of nephrolithiasis. Pediatr Nephrol. 2000;14(12):1102-4.

31. Resnick M, Pridgen DB, Goodman HO. Genetic predisposition to formation of calcium oxalate renal calculi. The New England journal of medicine. 1968;278(24):1313-8.
32. Oliveira B, Kleta R, Bockenhauer D, Walsh SB. Genetic, pathophysiological, and clinical aspects of nephrocalcinosis. American journal of physiology Renal physiology. 2016;311(6):F1243-F52.
33. Stechman MJ, Loh NY, Thakker RV. Genetic causes of hypercalciuric nephrolithiasis. Pediatr Nephrol. 2009;24(12):2321-32.
34. Acevedo-Arozena A, Wells S, Potter P, Kelly M, Cox RD, Brown SD. ENU mutagenesis, a way forward to understand gene function. Annual review of genomics and human genetics. 2008;9:49-69.
35. Tchekneva EE, Khuchua Z, Davis LS, Kadkina V, Dunn SR, Bachman S, et al. Single amino acid substitution in aquaporin 11 causes renal failure. J Am Soc Nephrol. 2008;19(10):1955-64.
36. Weiss MJ, Cole DE, Ray K, Whyte MP, Lafferty MA, Mulivor RA, et al. A missense mutation in the human liver/bone/kidney alkaline phosphatase gene causing a lethal form of hypophosphatasia. Proceedings of the National Academy of Sciences of the United States of America. 1988;85(20):7666-9.
37. Gorvin CM, Rogers A, Stewart M, Paudyal A, Hough TA, Teboul L, et al. N-ethyl-N-nitrosourea-Induced Adaptor Protein 2 Sigma Subunit 1 (Ap2s1) Mutations Establish Ap2s1 Loss-of-Function Mice. JBMR Plus. 2017;1(1):3-15.
38. Pfaffl MW. A new mathematical model for relative quantification in real-time RT-PCR. Nucleic acids research. 2001;29(9):e45.
39. Young MJ, Longley MJ, Li FY, Kasiviswanathan R, Wong LJ, Copeland WC. Biochemical analysis of human POLG2 variants associated with mitochondrial disease. Human molecular genetics. 2011;20(15):3052-66.
40. Vafai SB, Mootha VK. Mitochondrial disorders as windows into an ancient organelle. Nature. 2012;491(7424):374-83.
41. Hannan FM, Walls GV, Babinsky VN, Nesbit MA, Kallay E, Hough TA, et al. The Calcilytic Agent NPS 2143 Rectifies Hypocalcemia in a Mouse Model With an Activating Calcium-Sensing Receptor (CaSR) Mutation: Relevance to Autosomal Dominant Hypocalcemia Type 1 (ADH1). Endocrinology. 2015;156(9):3114-21.
42. Kujoth GC, Hiona A, Pugh TD, Someya S, Panzer K, Wohlgemuth SE, et al. Mitochondrial DNA mutations, oxidative stress, and apoptosis in mammalian aging. Science. 2005;309(5733):481-4.
43. Humble MM, Young MJ, Foley JF, Pandiri AR, Travlos GS, Copeland WC. Polg2 is essential for mammalian embryogenesis and is required for mtDNA maintenance. Human molecular genetics. 2013;22(5):1017-25.
44. Ronaghi M, Uhlen M, Nyren P. A sequencing method based on real-time pyrophosphate. Science. 1998;281(5375):363, 5.
45. McKenna A, Hanna M, Banks E, Sivachenko A, Cibulskis K, Kernytsky A, et al. The Genome Analysis Toolkit: a MapReduce framework for analyzing next-generation DNA sequencing data. Genome research. 2010;20(9):1297-303.
46. Town M, Jean G, Cherqui S, Attard M, Forestier L, Whitmore SA, et al. A novel gene encoding an integral membrane protein is mutated in nephropathic cystinosis. Nat Genet. 1998;18(4):319-24.

47. Goldfarb DS, Fischer ME, Keich Y, Goldberg J. A twin study of genetic and dietary influences on nephrolithiasis: a report from the Vietnam Era Twin (VET) Registry. Kidney international. 2005;67(3):1053-61.
48. Halbritter J, Baum M, Hynes AM, Rice SJ, Thwaites DT, Gucev ZS, et al. Fourteen monogenic genes account for 15% of nephrolithiasis/nephrocalcinosis. J Am Soc Nephrol. 2015;26(3):543-51.
49. Lee YS, Kennedy WD, Yin YW. Structural insight into processive human mitochondrial DNA synthesis and disease-related polymerase mutations. Cell. 2009;139(2):312-24.
